# Echogenicity Changes in Brainstem Raphe Detected by Transcranial Parenchymal Sonography and Clinical Characteristics in Parkinson's Disease

**DOI:** 10.3389/fneur.2020.00821

**Published:** 2020-08-07

**Authors:** Hong-Zhe Bei, Ju-Ping Chen, Cheng-Jie Mao, Ying-Chun Zhang, Jing Chen, Qiao-Qiao Du, Fei Xue, Pei-Cheng He, Hong Jin, Fu-Yu Wang, Chun-Feng Liu

**Affiliations:** ^1^Department of Neurology, The Third Affiliated Hospital of Inner Mongolia Medical University, Baotou, China; ^2^Department of Neurology and Suzhou Clinical Research Center of Neurological Diseases, The Second Affiliated Hospital of Soochow University, Suzhou, China; ^3^Department of Neurology, Changshu Hospital Affiliated to Nanjing University of Chinese Medicine, Changshu, China; ^4^Institutes of Neuroscience, Soochow University, Suzhou, China; ^5^Department of Ultrasound, The Second Affiliated Hospital of Soochow University, Suzhou, China; ^6^Department of Physical Examination Center, The Second Affiliated Hospital of Soochow University, Suzhou, China; ^7^Department of Neurology, Suqian First Hospital, Suqian, China

**Keywords:** Parkinson's disease, depression, anxiety, transcranial parenchymal sonography, brainstem raphe

## Abstract

**Background:** Decreased brainstem raphe (BR) echogenicity detected by transcranial parenchymal sonography (TCS) is associated with depression in psychiatric and neurologic diseases. However, previous studies focusing on the relationship between motor and non-motor symptoms and echogenicity changes in BR in patients with PD yielded controversial results.

**Objectives:** To investigate the relationship between echogenicity changes in BR detected by TCS and motor and a series of non-motor symptoms in patients with PD.

**Methods:** Consecutive PD patients were recruited from the Second Affiliated Hospital of Soochow University. Demographic information and Motor and non-motor symptoms for all subjects were collected. TCS was used to detect the echogenicity changes in BR in PD patients.

**Results:** One hundred and thirty-five consecutive patients with PD were enrolled in the study. The BR abnormal rate was significantly higher in PD patients with anxiety (*p* = 0.003) or depression (*p* = 0.022) than patients without. Spearman correlation analyses showed that Hamilton Rating Scale for Depression(HRSD) (*r* = 0.274, *p* = 0.002) and Parkinson's Disease Questionnaire 39-item(PDQ-39) (*r* = 0.208, *p* = 0.034) scores were positively correlated with abnormal BR echogenicity. Multivariate logistic regression analyses showed that HRSD and HAMA scores were associated with BR hypoechogenicity, the corresponding odds ratios (confidence intervals) were 1.07 (95% CI, 1.01–1.13) and 1.10(1.01–1.18), respectively. However, the PDQ-39 score was not associated with BR hypoechogenicity.

**Conclusion:** The abnormal reduction in BR echogenicity detected by TCS is associated with depression and anxiety, but not motor symptoms in PD patients.

## Introduction

Parkinson's disease (PD) is pathologically characterized by degeneration and loss of dopamine neurons in the substantia nigra (SN) and decreased dopamine content in the striatum, which result in motor symptoms such as tremor, rigidity and bradykinesia, and non-motor symptoms such as psychiatric symptoms, cognitive dysfunction, autonomic dysfunction, sleep disorder, and abnormal sensation. Transcranial parenchymal sonography (TCS), a type of non-invasive neuroimaging technology can detect brain parenchymal lesions directly *in vivo* and was first used in PD patients by Becker in 1995 (1). Abnormal hyperechogenicity of the SN is also considered to be a prodromal marker of PD. The sensitivity and specificity of SN hyperechogenicity for predicting PD are 82.4 and 82.5%, respectively (2). Recent studies have shown that patients with depression have abnormal brainstem raphe (BR) echogenicity (3–5). Combining SN hyperechogenicity with BR hypoechogenicity may be useful to detect individuals at risk for developing PD (6). The incidence of BR hypoechogenicity was much higher in PD patients with depression than patients without depression and controls (7, 8). Besides depression, reduced echogenicity of BR also indicated an increased risk of other non-motor symptoms in PD patients, such as urinary incontinence (9). However, previous studies focusing on the relationship between motor and non-motor symptoms and echogenicity changes in BR in patients with PD yielded controversial results. For example, Bouwmans et al. found no association between depression and hyperechogenic SN or hypoechogenic BR in PD patients (10).

In this study, TCS was used to detect the changes in BR echogenicity in PD patients whose motor and non-motor symptoms were comprehensively evaluated by the Unified Parkinson's Disease Rating Scale (UPDRS) and several non-motor symptom scales. We aimed to investigate the relationship between the changes in BR echogenicity and motor and a series of non-motor symptoms in PD patients.

## Materials and Methods

### Subjects

**Figure 1:**
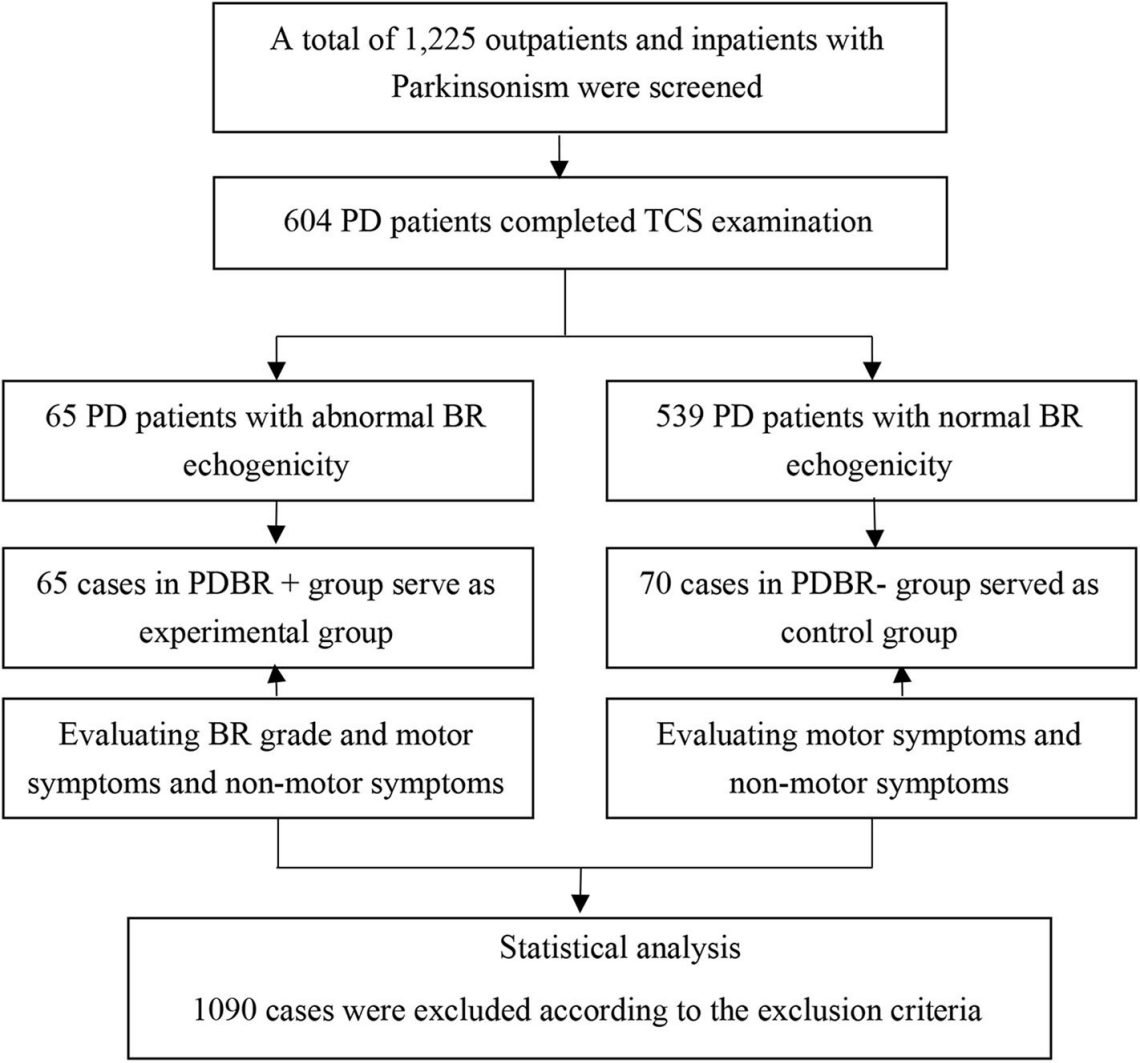
Flowchart of patient enrollment. This figure shows how the subjects enrolled in the study.

All PD subjects come from outpatient and hospitalized patients in the Second Affiliated Hospital of Soochow University from January 2011 to December 2015 and satisfied the UK Parkinson's Disease Society Brain Bank clinical diagnostic criteria (11). All subjects underwent TCS. Subjects were excluded if they had a secondary parkinsonism syndrome, deep brain stimulation, parkinson-plus syndrome, atypical parkinsonian syndrome, malignant neoplasm, epilepsy, or severe cardiopulmonary disease. Subjects who could not complete the motor or non-motor symptoms evaluation were also excluded. The flow chart shows the procedure for subject enrollment (Figure 1).

Demographic data, including age, gender, age at onset, disease duration, and detailed medical history were collected. All subjects were carefully evaluated by a movement disorder specialist. The UPDRS (12) and Hoehn & Yahr (H&Y) (13) scale were applied in all PD subjects during the “ON” medication state to evaluate motor symptoms. A Chinese version of the Montreal Cognitive Assessment (MoCA) (14) questionnaire (Beijing version) were used to evaluate the cognitive function of these patients. Neuropsychiatric symptoms were evaluated by the Hamilton Rating Scale for Depression (HRSD-24) (15)and the Hamilton Anxiety Scale (HAMA-14) (16). For HRSD-24, each item can range from 0 to 4 points. Patients with PD with HRSD-24 score ≥8 were defined as PD with depression. Parkinson's Disease Questionnaire 39-item (PDQ-39) (17) were also used in PD patients (Figure 1).

Calculation of a daily levodopa equivalent dose (LED) for each patient was based on theoretical equivalence to levodopa as follows: levodopa dose + levodopa dose × 1/3 if on entacapone + piribedil (mg) + pramipexole (mg) 100 + selegiline (mg) × 10 +amantadine (mg) + controlled release levodopa (mg) × 0.75.

Only one patient was on the treatment with antidepressants.

### Transcranial Parenchymal Sonography

A color-coded phased-array ultrasound system, equipped with a 2.5 MHz transducer (Sequoia 512, Siemens Medical Solutions USA, Inc. 4V1C transducer) was used to detect signals through the right and left temporal bone windows in the axial plane (18–21).

The midbrain was identified as a butterfly-shaped low-echogenic area, surrounded by the hyperechogenic basal cistern. SN is a hyperechogenic area with respect to surrounding structures. SN echogenic size measurements were performed on axial TCS scans automatically after manually encircling the outer circumference of the SN's echogenic area. Areas with SN echogenicity ≥0.20 cm^2^ on either side were classified as hyperechogenic (22).

**Figure 2:**
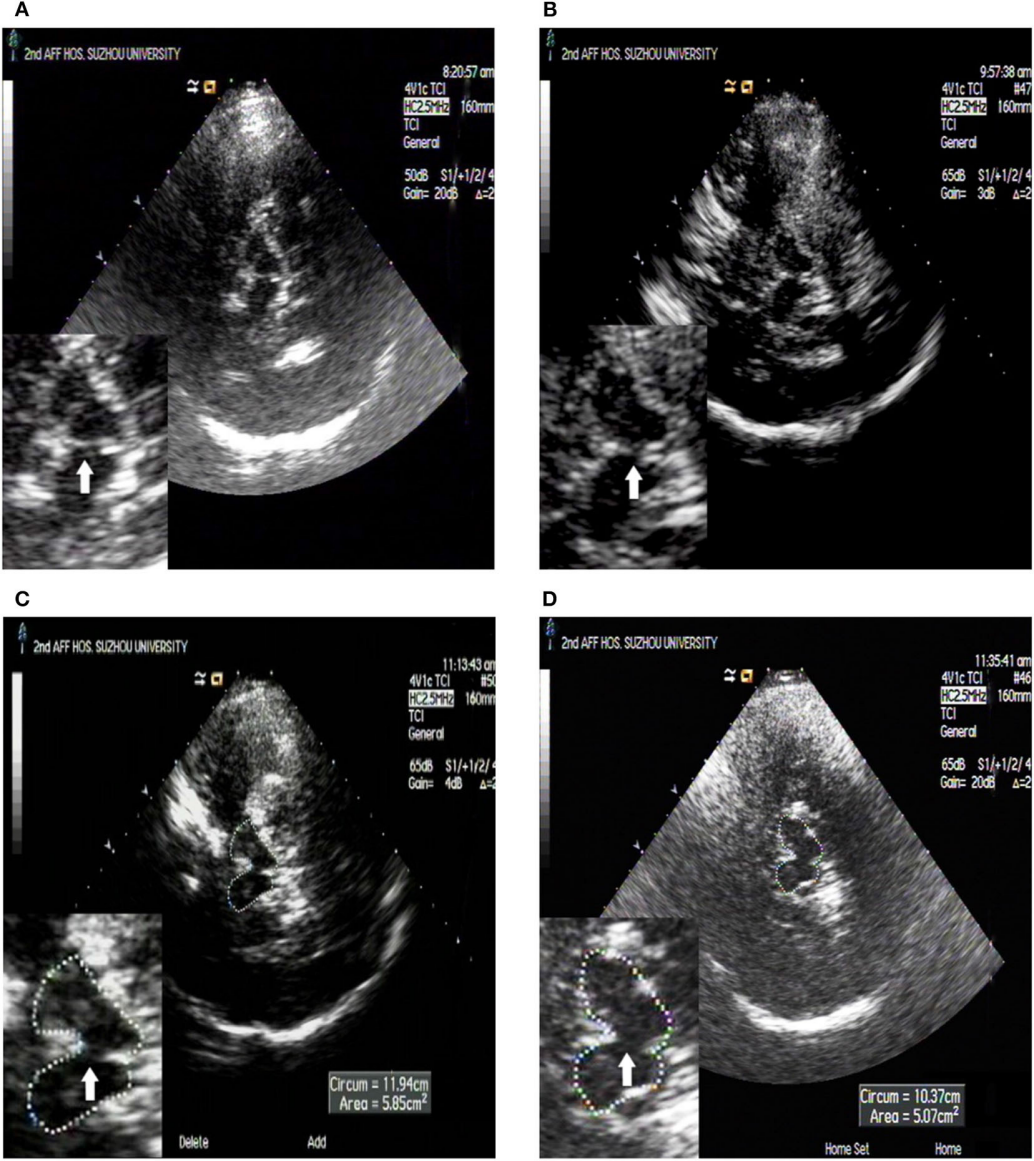
TCS images of BR echogenicity. BR semiquantitatively rated grade scale: the white arrow shows the BR. **(A)** Normal raphe, with the same echogenicity as the red nucleus according to previous recommendations; **(B)** Decreased raphe, echogenic raphe was decreased compared with the red nucleus but it was continuous; **(C)** Interrupted raphe, echogenic raphe was interrupted compared with the red nucleus; **(D)** Invisible raphe, echogenic raphe was not visible.

The BR was detected as a hyperechogenic continuous line in the middle of the midbrain with the same echogenicity as the red nucleus. The best images were selected for the study (20, 21). BR echogenicity was categorized according to current guideline recommendations on 2 grades of BR echogenicity (normal vs. reduced echogenicity) (20). Patients with BR echogenicity same as red nucleus were determined as BR echogenicity normal group, while patients with BR echogenicity as reduced, interrupt or not visible were determined as BR echogenicity abnormal group in this study (Figure 2).

All TCS assessments were performed by two experienced examiners who were blinded to the clinical data. Patients with different BR grades as rated by the two sonologists were excluded.

This study was approved by the ethics committee of our hospital and an informed consent was obtained from each patient.

### Statistical Analysis

Normally distributed continuous variables are presented as means ± standard deviations (SD), skewed distributed continuous variables are presented as median (interquartile range), and comparisons between two groups were performed by the Student's *t*-test or non-parametric test, respectively. Categorical variables are described as frequencies (percentages) and compared between groups using the Chi-square test. Bonferroni correction has been applied for multiple comparisons. Spearman rank correlation and multivariate logistic regression analysis were used to assess the correlation between BR echogenicity score and the motor and non-motor symptoms. All *p-*values were 2 tailed, and a significance level of 0.05 was used. Statistical analysis was conducted using SPSS version 21 (IBM SPSS, Chicago, IL, USA).

## Results

There were 652 PD patients undergoing the TCS examination. Forty-eight (7.36%, 48/652) PD patients were excluded due to insufficient transtemporal bone window. Six hundred four patients with PD completed the TCS examination. One hundred and thirty-five consecutive patients with PD were enrolled in this study, eventually.

### Demographic Data and Changes in BR Measured by TCS

Sixty-five (35 males and 30 females) patients had abnormal BR echogenicity and were aged 64.15 ± 8.42 years, age at onset was 60.13 ± 8.63 years, and disease duration was 46.5 (42.25) months. Seventy (48 males and 22 females) patients had normal BR echogenicity. The average age of the patients was 63.13 ± 9.36 years, age at onset was 59.66 ± 9.60 years, and disease duration was 33–39 months. No statistically significant differences were observed for gender, age, age at onset, disease duration, and education between the BR echogenicity normal group and abnormal group (Table 1).

**Table 1:** Demographic data of PD patients in the abnormal and normal BR echogenicity group.

Characteristics^*^	**Total** **(*n* = 135)**	**BR abnormal** **group** **(*n* = 65)**	**BR normal** **group** **(*n* = 70)**	***p*-value**
Male	83 (61.48)	35 (53.85)	48 (68.57)	0.079
Age, years	63.62 ± 8.90	64.15 ± 8.42	63.13 ± 9.36	0.506
Age at onset, years	59.88 ± 9.11	60.13 ± 8.63	59.66 ± 9.60	0.768
Disease duration, months	36.0 (22.0–60.0)	46.5 (22.5–63.0)	33.0 (22.0–60.0)	0.145
**Education**				
Illiteracy	25 (18.52)	14 (21.54)	11 (15.71)	0.709
Primary school	26 (19.26)	13 (20.00)	13 (18.57)	
Middle school	40 (29.63)	16 (24.62)	24 (34.29)	
High school	20 (14.81)	11 (16.92)	9 (12.86)	
University	24 (17.78)	11 (16.92)	13 (18.57)	
Daily levodopa-equivalent dose(mg)	262.5 (0–400.0)	300.0 (37.5–400.0)	250.0 (0–381.0)	0.314
Areas of SN hyperechogenicity	0.58 (0.39–0.99)	0.62 (0.49–1.06)	0.56 (0.39–0.93)	0.332
Number of SN hyperechogenicity (*n*,%)	(58, 43.0%)	(22, 33.8%)	(36, 51.4%)	0.039

* *Continuous variables are expressed as mean ± standard deviation or as median (interquartile range). Categorical variables are expressed as frequency (percent)*.

*SN, substantia nigra*.

### Changes in BR Echogenicity and Motor and Non-motor Symptoms in PD Patients

The HRSD (*Z* = 3.052, *p* = 0.002), HAMA (*t* = 2.472, *p* = 0.017), and PDQ-39 (*Z* = 2.117, *p* = 0.034) scores were higher in the BR abnormal group than in the BR normal group. The BR abnormal rate was significantly higher in PD patients with anxiety (*p* = 0.003) or depression (*p* = 0.022) than patients without. For multiple comparisons, the threshold for statistical significance after Bonferroni correction was set at *p* < 0.007 (correcting for 7 comparisons: 0.05/7 ≈ 0.007). HRSD was statistically significant after Bonferroni correction (*p* < 0.007) (Table 2).

**Table 2:** Comparison of motor and non-motor symptoms between the abnormal and normal BR echogenicity group.

Characteristics^*^	**Total** **(*n* = 135)**	**BR abnormal** **group** **(*n* = 65)**	**BR normal** **group** **(*n* = 70)**	***p*-value**
UPDRS II	10.61 ± 5.70	11.31 ± 6.10	9.97 ± 5.27	0.175
UPDRS III	23.06 ± 12.74	24.29 ± 13.15	21.91 ± 12.33	0.28
H-Y stage	2.0 (1.5–2.5)	2.0 (1.5–3.0)	2.0 (1.5–2.0)	0.106
MoCA	21.28 ± 4.88	21.32 ± 4.76	21.25 ± 4.99	0.945
HRSD	6.0 (2.0–12.0)	9.0 (4.0–15.0)	5.0 (1.0–8.0)	0.002
HRSD≥8	49 (36.3%)	30 (61.2%)	19 (38.8%)	0.022^†^
HRSD < 8	86 (63.7%)	35 (40.7%)	51 (59.3%)	
HAMA	6.52 ± 6.69	9.09 ± 8.88	5.11 ± 4.63	0.017
HAMA≥7	63 (46.7%)	39 (61.9%)	24 (38.1%)	0.003^#^
HAMA < 7	72 (53.3%)	26 (36.1%)	46 (63.9%)	
PDQ-39	15.0 (6.0–39.0)	15.0 (6.0–45.0)	12.0 (9.0–45.0)	0.034

* *Continuous variables are expressed as mean ± standard deviation or as median (interquartile range). Categorical variables are expressed as frequency (percent)*.

† *Comparisons of the abnormal BR echogenicity rate between depression group and non-depression group*.

# *Comparisons of the abnormal BR echogenicity rate between anxiety group and non-anxiety group*.

*UPDRS II, second part of the Unified Parkinson Disease Rating Scale score; UPDRS III, third part of the Unified Parkinson Disease Rating Scale score; H-Y stage, Hoehn-Yahr stage; MoCA, Montreal Cognitive Assessment; HRSD, Hamilton Rating Scale for Depression; HAMA, Hamilton Anxiety Scale; PDQ-39, Parkinson's Disease Questionnaire-39*.

### Spearman Rank Correlation and Multivariate Logistic Regression Analysis Between BR Echogenicity and the HRSD, HAMA, and PDQ-39 Score

Spearman rank correlation analysis revealed that abnormal BR echogenicity was positively correlated with the HRSD and PDQ-39 score, with low correlation coefficients (*r* = 0.274, *p* = 0.002 for HRSD; *r* = 0.208, *p* = 0.034 for PDQ-39). Spearman rank correlation analysis suggested a marginally statistical significant association between BR hypoechogenicity and HAMA score (*r* = 0.201, *p* = 0.047). After adjusting age, gender, age at onset of PD, education, disease duration, LED, UPDRS II, UPDRS III, and H-Y stage, only HRSD, and HAMA score were associated with BR hypoechogenicity with ORs 1.07 (95% CI, 1.01–1.13), and 1.10 (1.01–1.18), respectively (Tables 3, 4).

**Table 3:** Spearman rank correlation analysis between BR echogenicity and depression, anxiety and PDQ-39.

**Variables**	***r*-value**	***p-*value**
HRSD	0.274	0.002
HAMA	0.201	0.047
PDQ-39	0.208	0.034

*PDQ-39, Parkinson's Disease Questionnaire-39; HRSD, Hamilton Rating Scale for Depression; HAMA, Hamilton Anxiety Scale*.

**Table 4:** Multivariate logistic regression analysis of depression, anxiety, and PDQ-39 with BR hypoechogenicity.

**Variables**	**Odds ratio (95% confidence interval)**	***p-*value**
**HRSD, per 5 score increase**		
Unadjusted model	1.45 (1.13–1.86)	0.004
Adjusted model	1.07 (1.01–1.13)	0.022
**HAMA, per 5 score increase**		
Unadjusted model	1.59 (1.12–2.26)	0.009
Adjusted model	1.10 (1.01–1.18)	0.021
**PDQ-39, per 5 score increase**		
Unadjusted model	1.10 (1.00–1.21)	0.05
Adjusted model	1.08 (0.94–1.25)	0.260

*Adjusted model included age, gender, age at onset of PD, education, disease duration, daily levodopa-equivalent dose, UPDRS II, UPDRS III, and Hoehn-Yahr stage*.

## Discussion

It has been proved that TCS is reliable and sensitive in detecting basal ganglia abnormalities, e.g., of SN in PD. Many studies focusing on the echogenicity of the SN and PD diagnosis or clinical characteristics. Hyperechogenicity of SN has high diagnostic accuracy in the diagnosis of PD patients from healthy controls (23). In addition, PD patients with depression had marked SN hyperechogenicity and reduced echogenicity of BR indicating SN hyperechogenicity combined with reduced echogenicity of BR might be useful to detect individuals at risk for developing PD (24). Some studies have demonstrated a correlation between abnormal BR echogenicity and depression (3). BR hypoechogenicity is more common in certain types of PD, such as glucocerebrosidase gene(GBA) mutations related to PD (25). The present study aims to investigate the relationship between changes in BR echogenicity and motor and a series of non-motor symptoms, such as depression, anxiety, and cognition in PD patients.

Spearman rank correlation analysis showed weak correlations between HRSD and PDQ-39 scores and the reduction in BR echogenicity, and multivariate logistic regression revealed that HRSD and HAMA scores were associated with BR hypoechogenicity in the adjusted model. No association was found between PDQ-39 scores and BR hypoechogenicity. Cho et al. found that decreased BR echogenicity was much higher in PD patients with depression (7). PD patients with depression and patients with depression only showed a significantly higher presence of abnormal BR than those without depression and healthy controls (6). A significant direct relationship was also found between the BDI score and BR hypoechogenicity (8). Our study confirmed the relationship between BR hypoechogenicity and depression. BR alterations in TCS may be a biomarker for depression and apathy in PD patients (26). Decreased BR echogenicity indicates morphological alterations in the midbrain which is involved in the pathogenesis of depression not only in PD patients with depression but also in unipolar depression patients (6, 8, 25). Abnormal BR echogenicity could also be seen in *de novo* PD patients with depression, which could also be found in both control and PD groups without depression (7). These TCS findings support the hypothesis of a pathogenetic link between depression and PD (9). However, conflicting findings have also been reported (6, 10). Bouwmans et al. found no association between depression and hyperechogenic SN or hypoechogenic BR in PD patients (10). The main reason for the difference may be the disease severity. We noticed that the patients included in their study were early PD patients. UPDRS III score of patients was significantly lower than the score of our patients and others (10).

Furthermore, spearman rank correlation analysis suggested a marginal statistical significance association between BR hypoechogenicity and HAMA score. Besides the dopamine system, neurodegeneration of neurons involved several other neurotransmitter systems, such as the norepinephrine system, serotonin system, and acetylcholine system. BR is the main source of serotonin in the prefrontal cortex. Changes in BR echogenicity may reflect a decline in the function of the serotonin system (27). The overlap of widespread dysfunction of the limbic system and complex neurotransmission abnormalities in PD patients with depression and anxiety may explain the correlation between reduced echogenicity of BR and anxiety (28).

In this study, we noticed that only 48 (7.36%, 48/652) PD patients were excluded due to insufficient transtemporal bone window, which is remarkably lower compared to other studies in the Asian population (7, 29), but consistent with our previous studies (30–32). This may be because of the 2.5 MHz transducer we used (Sequoia 512, Siemens Medical Solutions USA, Inc. 4V1C transducer), which was compared with other transducers and showed the best penetration.

There were some limitations of the study. This was a cross-sectional study, and we were unable to draw a conclusion about the relationship between changes in BR echogenicity and the clinical manifestations of PD as the disease progressed. Also, the sample size in this study was relatively small, and studies with a large number of PD patients from multiple centers are needed to confirm the results.

In summary, an abnormal reduction in BR echogenicity detected by TCS is associated with depression and anxiety, but not motor symptoms in PD patients.

## Data Availability Statement

The raw data supporting the conclusions of this article will be made available by the authors, without undue reservation.

## Ethics Statement

The studies involving human participants were reviewed and approved by the ethics committee of the Second Affiliated Hospital of Soochow University. The patients/participants provided their written informed consent to participate in this study.

## Author Contributions

H-ZB, J-PC, and C-JM designed the study, collected the data, and drafted the manuscript. Y-CZ, JC, FX, P-CH, HJ, and F-YW collected the data. Q-QD analyzed the data. C-FL designed the study. All authors contributed to the article and approved the submitted version.

## Conflict of Interest

The authors declare that the research was conducted in the absence of any commercial or financial relationships that could be construed as a potential conflict of interest.
